# Efficacy and safety of modified bleomycin administration with EP chemotherapy in adult male patients with germ cell tumors: a retrospective study

**DOI:** 10.1186/s12935-025-03774-2

**Published:** 2025-04-16

**Authors:** Ditian Shu, Riqing Huang, Meiting Chen, Haifeng Li, Xin An, Cong Xue, Anqi Hu, Fangjian Zhou, Kai Yao, Zhuowei Liu, Yanxia Shi

**Affiliations:** 1 State Key Laboratory of Oncology in South China, Guangdong Provincial Clinical Research Center for Cancer, Sun Yat-Sen University Cancer Center, Guangzhou, 510060 People’s Republic of China; 2 Department of Medical Oncology, Sun Yat-Sen University Cancer Center, No. 651 Dongfeng East Road, Guangzhou, 510060 People’s Republic of China; 3 Department of Urology, Sun Yat-Sen University Cancer Center, No. 651 Dongfeng East Road, Guangzhou, 510060 People’s Republic of China

**Keywords:** Germ cell tumors, Modified chemotherapy, Pulmonary toxicity, BEP

## Abstract

**Background:**

Given the substantial inconvenience caused by weekly bleomycin administration, we initiated a study to evaluate the efficacy and toxicity of the modified bleomycin combined with EP (modified-BEP) regimen in Chinese adult male patients with germ cell tumors (GCTs).

**Methods:**

We conducted a retrospective analysis of 274 adult male GCT patients treated with modified-BEP at the Sun Yat-sen University Cancer Center between 2005 and 2022. The regimen involved a tri-weekly 5-day schedule with 30 IU modified bleomycin (administered on days 1, 3, and 5), 20 mg/m2 cisplatin (days 1–5), and 100 mg/m2 etoposide (days 1–5). The survival rates and safety profiles of the patients were analyzed.

**Results:**

Among the patients, 42 patients received BEP in adjuvant setting, while 232 were treated with BEP in curative setting. With a median follow-up of 41.03 months among the curative patient population, the 5-year progression-free survival (PFS) rate was 79.33%, and the 5-year overall survival (OS) rate was 86.26%. Stratified by the International Germ Cell Cancer Collaborative Group (IGCCCG) prognostic groups, the 5-year OS rates of the good, intermediate, and poor risk groups were 99.05%, 92.84%, and 55.96% (P < 0.0001), respectively. Favorable responses, including complete remission and partial response with negative tumor markers, were achieved in 91.07% of good-risk, 84.13% of intermediate-risk, and 52.63% of poor-risk patients, with a significant difference (*P* < 0.0001). Multivariate analysis indicated that non-seminoma, poor risk group, mediastinal primary tumor, and Eastern Cooperative Oncology Group (ECOG) 2 status were significantly associated with inferior PFS. In the entire cohort, major grade 3—4 adverse events included neutropenia (38.69%), anemia (4.74%), thrombocytopenia (5.11%), and febrile neutropenia (6.2%), with no death due to pulmonary toxicity.

**Conclusion:**

The modified-BEP regimen showed an effective and tolerable treatment alternative for adult male GCT patients in China, offering greater convenience compared to the standard BEP regimen.

## Introduction

Germ cell tumors (GCTs) predominantly affect younger males between 15 and 40 years, accounting for an estimated 74,500 new global cases in 2020 [1]. In China, the annual incidence of testicular cancer is 4,000 cases, with a mortality of 1,000 individuals [2]. Patients with metastatic GCTs are stratified into good, intermediate, and poor risk categories based on the International Germ Cell Cancer Collaborative Group (IGCCCG) risk criteria, relying on pretreatment clinical features [3]. These risk categories are directly correlated with long-term complete response (CR) rates to chemotherapy, with estimated CR rates of approximately 90%, 75%, and 40% for good, intermediate, and poor risk patients, respectively [3].

The standard first-line chemotherapy for these patients, the bleomycin, etoposide, and cisplatin (BEP) regimen [4], involves a 30 IU intravenous (IV) bolus of bleomycin weekly, either on days 1, 8, and 15 or on days 2, 9, and 16, combined with etoposide and cisplatin over a 21-day cycle. This regimen yields favorable treatment outcomes. Most patients classified as having a good prognosis according to the IGCCCG criteria for metastatic GCTs are cured with 3 cycles of BEP [5]. However, the weekly administration of bleomycin poses significant challenges; it not only imposes considerable inconvenience on patients but also increases their financial burden. This frequent treatment schedule can significantly disrupt patients'daily activities and occupational responsibilities, adversely affecting their quality of life and potentially leading to poor treatment compliance.

Recognizing these challenges, recent decades have seen clinical trials aimed at refining the BEP regimen without compromising cure rates [6–8]. In our center, bleomycin is typically administered on days 1, 3, and 5 instead of the standard dosing (days 1, 8, and 15) based on our experience using BEP-based therapy over the past 2 decades. However, real-world data on such modified regimens, particularly among Asian populations, remains scarce. Therefore, this study aims to fill this gap by presenting retrospective data on the efficacy and toxicity of our modified BEP regimen in a substantial cohort of adult Chinese patients with GCTs.

## Materials and methods

### Patients and treatment

From December 2005 to November 2022, 274 adult patients with GCTs were treated with modified BEP at Sun Yat-sen University Cancer Center (SYSUCC). The study protocol was approved by the ethical committee of SYSUCC (approval number B2022 - 268–01). Eligible patients had histologically confirmed testicular, retroperitoneal, or mediastinal primary GCTs, received at least one dose of modified BEP with an available response assessment, had received no prior chemotherapy, and had adequate cardiac, bone marrow, and hepatic function apart from organ function affected by the disease. The diagnosis of GCTs was performed by experienced pathologists at SYSUCC. The stage at diagnosis was assessed using the American Joint Committee on Cancer’s Cancer Staging Manual, 8 th edition. The IGCCCG prognostic classification was used for allocating patients to risk categories [3].

The modified BEP regimen consisted of a triweekly 5-day hospitalization schedule for 5 days. This regimen included administering 30 IU of bleomycin intravenously on days 1, 3, and 5, cisplatin at a dose of 20 mg/m2via intravenous drip over 3 h from days 1 to 5, and etoposide at 100 mg/m2 administered over 60 min via intravenous drip on the same days. The indications for BEP adjuvant therapy were specifically targeted at marker-negative patients in clinical stages I and IIA post-retroperitoneal lymph node dissection (RPLND), with treatment commencing post-surgery. In the context of marker-positive stages IIA to IIIC, BEP was considered a curative treatment. Treatment cycles typically consisted of 2 cycles of adjuvant therapy post-RPLND for non-seminomas, 3 cycles for good-risk patients and those diagnosed with seminoma, and 4 cycles for patients classified as intermediate or poor-risk. All patients had a creatinine clearance > 60 mL per minute. Additionally, granulocyte colony-stimulating factor (G-CSF) was routinely administered via subcutaneous injection on day 6 of each cycle for the majority of patients.

The data reviewed included the patient demographic information, tumor characteristics, standard laboratory tests, computed tomography (CT) scans of the whole body, and the treatment regimens applied. The chemotherapy regimens included primarily etoposide, cisplatin, and bleomycin based on the patient’s performance state and renal function.

### Toxicity evaluation

Adverse events (AEs) were graded according to the Common Terminology Criteria for Adverse Events (version 5.0). Toxicity was assessed after each cycle. The relative frequency of each AE, considered as possibly, probably, or likely related to chemotherapy, was estimated as the proportion of all toxicity-evaluable cycles in which toxicity was observed. In the absence of a reliable diagnostic tool, we identified all cases of bleomycin-induced lung toxicity ranging from fibrosis changes, noted on chest X-ray or chest CT scan, to dyspnea requiring treatment with steroids. Pulmonary toxicity was assessed through a chest CT scan and clinical monitoring.

### Response assessment

Response assessment was carried out following every other cycle. The disease was also evaluated using Response Evaluation Criteria In Solid Tumours (RECIST) version 1.1 for response assessment. Complete remission (CR) to chemotherapy alone was defined as either radiographic resolution of disease or surgical resection of residual disease revealing necrosis or teratoma but no viable GCT, and normal serum human chorionic gonadotropin and alpha-fetoprotein for a minimum of 4 weeks. A CR to chemotherapy plus surgery requires resection of all residual masses, in which residual viable GCT is identified in at least one site. Partial response (PR) was defined as a greater than 30% decrease in bidimensional tumor measurements (PR-negative with normalization of previously elevated tumor markers; PR-positive without complete normalization). All CR and PR-negative are considered favorable responses. If elevated markers were the only evidence of disease, a decrease of at least 90% was required for a PR. Progressive disease (PD) was defined as a greater than 20% increase in bidimensional tumor measurements, an increase in tumor markers of more than 50%, or the appearance of new lesions. Levels of serum tumor markers were measured every 2–3 weeks. All responses as well as the diagnosis of stable disease were confirmed after a 4-week interval.

### Statistical analysis

The study population for all analyses included patients enrolled in the study who had received at least one dose of modified BEP. Patient characteristics, treatment administration, antitumor activity, and safety were summarized using descriptive statistics. Survival was measured from the initiation of therapy until death. The objective response rate (ORR), progression-free survival (PFS), overall survival (OS), type, incidence, severity, seriousness, relationship to study medications of AEs, and laboratory abnormalities were also analyzed. A cutoff date of February 17 th, 2023, was established for analyzing data for this report. OS and PFS were calculated from the initiation of the treatment to death and to progression or death, respectively. OS, and PFS rates were estimated using Kaplan-Meier analyses with SPSS 25.0 software (SPSS Inc., Chicago, IL, USA) and R version 4.2.2.

## Results

### Patient characteristics

A total of 274 patients were consecutively enrolled and treated (Table 1). Of these, 42 patients received BEP in adjuvant setting, while 232 were treated with BEP in curative setting. The median age for all patients at the initiation of treatment was 32 years (range: 18 to 62 years), with 14 patients over 50 years old. Most of the patients (87.59%) had an Eastern Cooperative Oncology Group performance status of 0 at the initiation of modified BEP. Among the cohort, 244 patients had received primary surgery, and 74 underwent RPLND. The histology was nonseminoma in 174 patients (63.5%) and pure seminoma in 100 patients (36.5%). The primary tumor sites were the testis in 201 patients (73.36%), the retroperitoneum in 34 patients (12.41%), and the mediastinum in 39 patients (14.23%). According to IGCCCG prognostic factor-based staging, 120 patients (43.80%) were classified as having a good prognosis, 63 patients (22.99%) as intermediate, and 57 patients (20.80%) as poor. Patients received a median of 4 cycles (range: 1 ~ 4) of modified BEP, with the total bleomycin dosage for each patient not exceeding 400IU.

**Table 1:** Patient characteristics

Characteristic	Overall, N = 274 (%)	Adjuvant, N = 42(%)	Curative, N = 232 (%)
Age			
Median (Range)	32(18—62)	36(18—62)	31(18–60)
ECOG			
0	238 (86.86%)	41 (97.62%)	197(84.91%)
1	30 (10.95%)	1 (2.38%)	29(12.5%)
2	6(2.19%)	0 (0.00)	6 (2.59%)
Smoking history			
No	218 (79.56%)	34 (80.95%)	184 (79.31%)
Yes	56 (20.44%)	8 (19.05%)	48 (20.69%)
Histology			
Seminoma	100 (36.50%)	24 (57.14%)	76 (32.76%)
Non seminoma	174 (63.50%)	18 (42.86%)	156 (67.24%)
Primary site			
Testis	201 (73.36%)	42 (100.00%)	159 (68.53%)
Retroperitoneum	34 (12.41%)	0 (0.00%)	34 (14.66%)
Mediastinum	39 (14.23%)	0 (0.00)	39 (16.81%)
Metastatic sites			
Abdominal lymph nodes	155 (56.57%)	4 (9.52%)	151 (65.09%)
Mediastinal lymph nodes	22 (8.03%)	0 (0.00)	22 (9.48%)
Supraclavicular lymph nodes	26 (9.49%)	0 (0.00)	26 (11.21%)
Lung metastases	70 (25.55%)	0 (0.00)	70 (30.17%)
Liver metastases	11 (4.01%)	0 (0.00)	11 (4.74%)
Bone metastases	9 (3.28%)	0 (0.00)	9 (3.88%)
Brain metastases	5 (1.82%)	0 (0.00)	5 (2.16%)
Other	20 (7.30%)	0 (0.00)	20 (8.62%)
Presence of NPVM			
Yes	37 (13.50%)	0 (0.00)	37 (15.95%)
No	237 (86.50%)	42 (100.00%)	195 (84.05%)
IGCCCG prognostic groups			
Good	120 (43.80%)	8 (19.05%)	112 (48.28)
Intermediate	63 (22.99%)	0 (0.00)	63 (27.16)
Poor	57 (20.80%)	0 (0.00)	57 (24.57)
Serum tumor markers			
S0	91 (33.21%)	42 (100.00%)	49 (21.12%)
S1	84 (30.66%)	0 (0.00)	84 (36.21%)
S2	71 (25.91%)	0 (0.00)	71 (30.60%)
S3	28 (10.22%)	0 (0.00)	28 (12.07%)
Total	274(100.00)	42(15.33%)	232((84.67%)

*NPVM* Nonpulmonary visceral metastases

### Efficacy outcome

With 232 patients in the curative setting, the median duration of follow-up at the data cut-off for the analysis was 41.03 months (95% CI 32.66–49.40). The ORR in the curative setting was 88.79%. The ORRs of the good, intermediate, and poor risk groups were 92.86%, 95.24%, and 73.68%, respectively (*P* = 0.0002). Favorable responses (complete remission and partial response with tumor marker negative) were achieved in 102 (91.07%) patients in the good-risk group, 53 (84.13%) in the intermediate-risk group, and 30 (52.63%) in the poor-risk group, with a significant difference (*P* < 0.0001). Early relapse occurred in 5.36%, 11.11%, and 45.61% of the good-, intermediate-, and poor-risk groups, respectively, while only 3 patients in the poor-risk group showed late relapse (more than two years after the end of first-line modified BEP treatment). In the entire curative patient population, 23 patients died from the progression of disease (Table 2).

**Table 2:** Summary of clinical responses in 232 patients with curative objective

	All, n (%)	Good, n (%)	Intermediate, n (%)	Poor, n (%)	*P* value
Best overall response, n (%)					
CR	116 (50.88%)	79(70.54%)	28(44.44%)	9(15.79%)	< 0.0001
PR-negative	69 (30.26%)	23(20.54%)	25(39.68%)	21(36.84%)	
PR-positive	21 (9.21%)	2(1.79%)	7(11.11%)	12(21.05%)	
SD	17 (7.46%)	4(3.57%)	2(3.17%)	11(19.3%)	
PD	5 (2.19%)	0	1(1.59%)	4(7.02%)	
NE	4(1.46%)	4(3.57%)	0	0	
ORR	206 (88.79)	104 (92.86)	60 (95.24)	42 (73.68)	0.0002
CR and PR-negative	185 (79.74)	102 (91.07)	53 (84.13)	30 (52.63)	< 0.0001
PFS status					
No progression	190 (81.90)	106 (94.64%)	56 (88.89%)	28 (49.12%)	< 0.0001
Early relapse	39 (16.81)	6 (5.36%)	7 (11.11%)	26 (45.61%)	
Late relapse*	3 (1.29)	0	0	3 (5.26%)	
OS status					
Alive	209 (90.09)	111 (99.11%)	59 (93.65%)	39 (68.42%)	< 0.0001
Death in the first 3 yrs	21 (9.05)	0	0	2 (3.51%)	
Death after 3 yrs	2 (0.86)	1 (0.89%)	4 (6.35%)	16 (28.07%)	
Follow-up (months)					
Median	41.03	30.03	49.8	45.03	–
95% CI	32.66–49.40	40.02–55.89	50.08–74.1	47.93–60.09	–

*CR* complete remission, *PR*-*negative*, partial response with tumor marker negative, *PR-positive* partial response with tumor marker positive, *PD* progressed disease, *SD* stable disease, *NE* nonevaluable, *ORR* objective response rate, *PFS* progression free survival; *OS* overall survival

Late relapse is defined as recurrence more than two years after end of first-line modified BEP chemotherapy

**Fig. 1:**
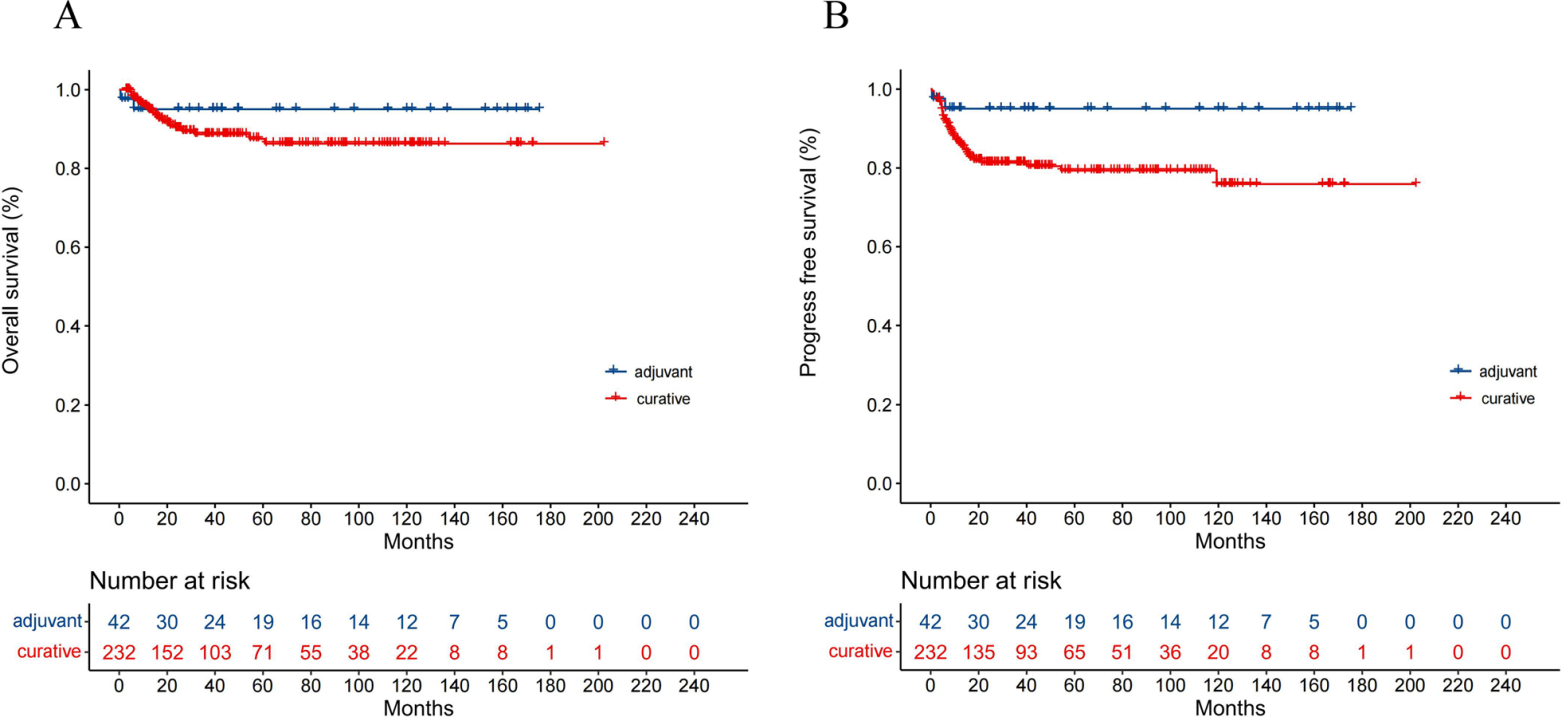
Survival probabilities according to treatment purpose for (**A**) overall survival (OS) and (**B**) progression-free survival (PFS)

**Fig. 2:**
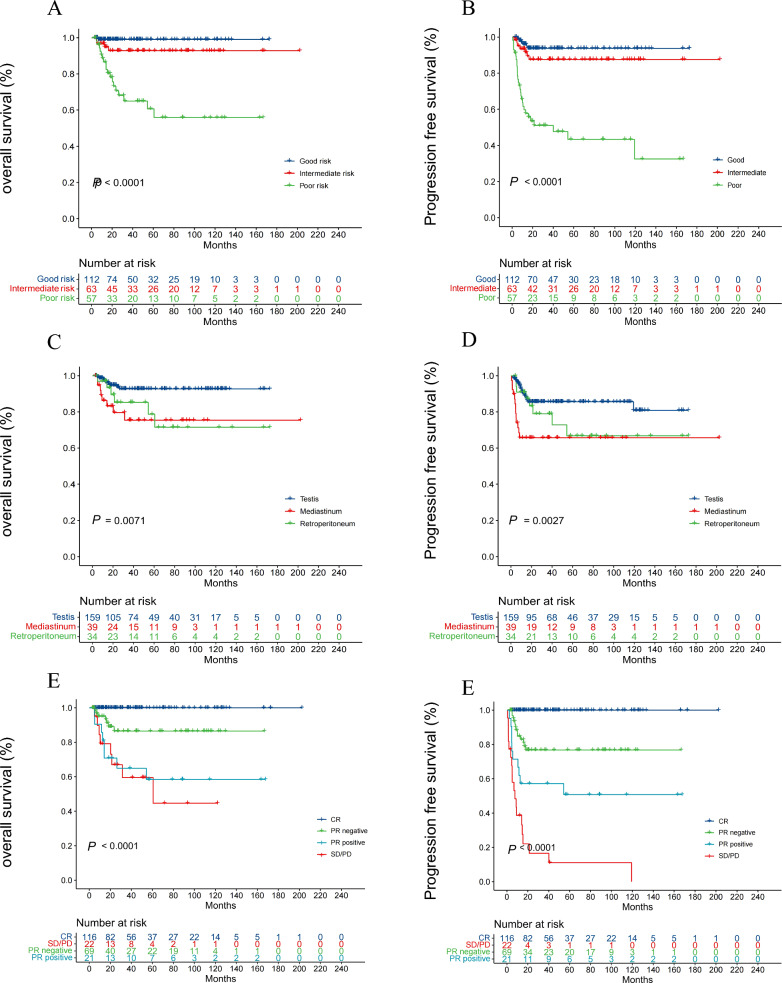
Survival probabilities according to IGCCCG prognostic groups for (**A**) OS and (**B**) PFS; survival probabilities stratified by primary site for (**C**) OS and (**D**) PFS; survival probabilities stratified by treatment response for (**E**) OS and (**F**) PFS

The median PFS and OS were not reached in the entire patient population. In the patients in the adjuvant setting, two died of heart disease during modified BEP treatment, and the 5-year OS rate and 5-year PFS rate among them were both 95.1% (Fig. 1A, Fig. 1B). The 5-year OS rate and 5-year PFS rate among the curative patient population were 86.26% and 79.33%, respectively (Fig. 1A, B). The 5-year OS rates of the good, intermediate, and poor risk groups were 99.05%, 92.84%, and 55.96%, respectively (*P* < 0.0001) (Fig. 2A). The PFS at 5 years of the good, intermediate, and poor risk groups were 93.75%, 87.57%, and 43.38%, respectively (*P* < 0.0001), and the medium PFS of the poor risk group was 40.07 months (Fig. 2B). When the curative patient population was stratified according to the primary site, the 5-year OS rates of the testis, mediastinum and retroperitoneum were 92.74%, 75.40%, and 71.52%, respectively (*P* < 0.0001) (Fig. 2C), and the 5-year PFS rates were 85.58%, 65.69%, and 66.78%, respectively (*P* < 0.0001) (Fig. 2D). The OS and PFS rates of patients stratified by treatment response are displayed in Fig. 2E and Fig. 2F. Patients who achieved PR with negative tumor markers after modified BEP treatment showed superior survival to patients who reached PR with positive tumor markers. No tumor progression or death occurred in the group of patients who achieved CR.

### Correlative analyses

**Fig. 3:**
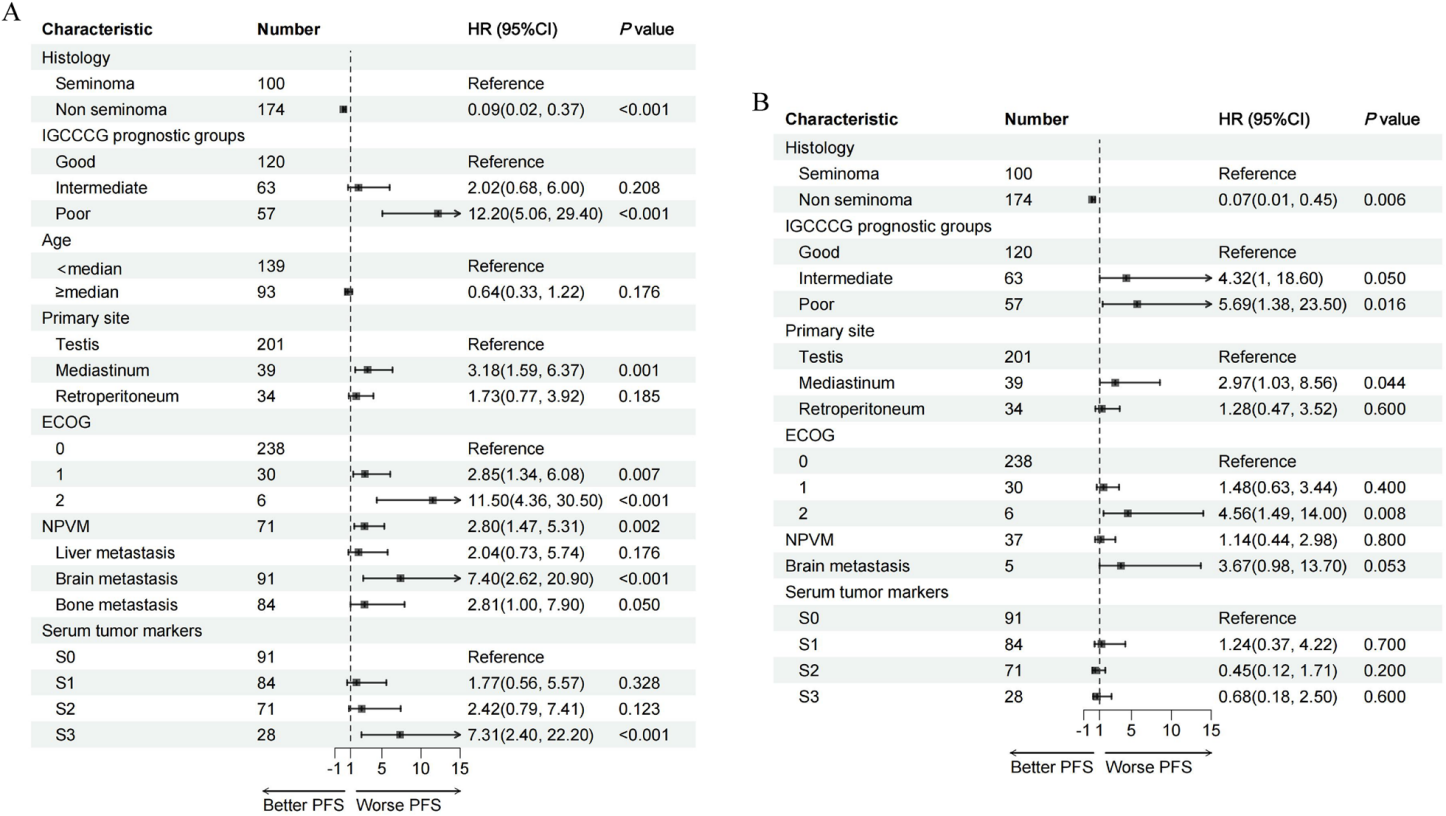
(**A**) PFS in univariable Cox regression analysis and (**B**) PFS in multivariable Cox regression analysis

To assess the factors that affect the PFS of modified BEP, we conducted comprehensive univariable and multivariate analyses (Fig. 3). The univariate analysis revealed that non-seminoma, poor risk group, mediastinal primary tumor, and higher Eastern Cooperative Oncology Group (ECOG) performance status, nonpulmonary visceral metastases (NPVM), brain metastasis, and elevated serum tumor markers were significantly associated with inferior PFS. Subsequently, multivariate analysis was performed to identify independent predictors of PFS, adjusting for potential confounders. Multivariate analysis indicated that non-seminoma, poor risk group, mediastinal primary tumor, and ECOG 2 status were significantly associated with inferior PFS.

### Safety profile

All patients were assessable for toxicity (Table 3). The principal AEs were hematological and gastrointestinal events. Regarding hematologic toxicity, the most common grade ≥ 3 treatment-related adverse events were neutropenia in 106 patients (38.69%), febrile neutropenia in 17 patients (6.2%), anemia in 13 patients (4.74%), and thrombocytopenia in 14 patients (5.11%). Regarding nonhematologic toxicity, 80 patients (29.2%) had grade 1/2 nausea, 77 patients (28.1%) experienced grade 1/2 vomiting, and 55 patients (20.07%) had grade 1/2 elevated transaminases. Bleomycin-related pulmonary toxicity was rigorously monitored in our study. Among the participants, 90 patients (32.85%) were followed for over five years, and 166 patients (60.58%) for more than two years. Four patients developed lung changes attributable to bleomycin, of which one was symptomatic. This patient experienced grade 3 pulmonary toxicity (interstitial pneumonitis and respiratory failure) six months post-treatment, and completely resolved after receiving 2 months of careful supportive treatment at a local hospital, although the specifics of the treatment remain unknown. Importantly, there were no deaths attributable to pulmonary toxicity in this cohort.

**Table 3:** Chemotherapy-related toxicity in all patients

Toxicity	Any Grade, n (%)	G3-G4, n (%)
Any adverse event	239(87.23%)	123(44.89%)
Hematologic		
Neutropenia	159(58.03%)	107(39.05%)
Febrile neutropenia	18(6.57%)	18(6.57%)
Anemia	132(48.18%)	13(4.74%)
Thrombocytopenia	40(14.6%)	14(5.11%)
Nonhematologic		
Dyspepsia	77(28.1%)	0
Vomiting	46(16.79%)	0
Nausea	80(29.2%)	0
Mucositis	7(2.55%)	0
Diarrhea	7(2.55%)	0
Pneumonia	4(1.46%)	1(0.37%)
Rash	3(1.09%)	0
Alopecia	18(6.57%)	2(0.73%)
Fatigue	8(2.92%)	0
Elevated transaminases	58(21.17%)	2(0.73%)
Serum creatinine increased	18(6.57%)	0

In the entire patient population, most patients showed good compliance, with only one patient refusing to continue modified-BEP chemotherapy because of febrile neutropenia. In addition, eight patients had prolonged modified BEP chemotherapy intervals due to hematological toxicity and elevated transaminases, and nine patients underwent chemotherapy dose reductions of 20–25% due to grade 3–4 hematological toxicity.

## Discussion

Currently, cisplatin-based combination chemotherapy achieves an estimated cure rate of 83% in patients with metastatic GCT [9]. Given an anticipated cure rate in excess of 90% of patients with IGCCCG good-risk disease [5, 10], standard regimens should prioritize both convenience and effectiveness. In this large, single-institution retrospective analysis that spanned 2 decades and included 274 patients from the Chinese population, we demonstrated that the modified BEP regimen maintains efficacy while offering greater convenience. For GCT patients eligible for chemotherapy with a BEP regimen, our modified triweekly 5-day BEP schedule presented a safe toxicity profile, with no deaths attributed to pulmonary toxicity, and showed promising response rates and survival outcomes. Additionally, our modified regimen provides an alternative that could be particularly beneficial to patients who may face challenges with travel and logistical burdens.

Regarding survival outcomes, an indirect comparison with the IGCCCG Update Model, which was published in 2021 and includes data from 9728 males with metastatic nonseminomatous GCTs, found that the 5-year PFS and 5-year OS rates in patients with poor risk were 54% and 67%, respectively [11]. In the present study, our survival rates were inferior to previously published data, with 5-year PFS and 5-year OS rates in patients with poor risk being 43.38% and 55.96%, respectively. The most important reason for this inconsistency may be related to the study population. In the updated model of IGCCCG, the primary sites of retroperitoneum and mediastinum accounted for 3.4% and 3.7%, respectively, whereas we observed much higher values for the primary sites retroperitoneum and mediastinum in our study, accounting for 12.41% and 14.23%, respectively. It can also be seen from our data that the survival of patients with primary retroperitoneum and mediastinum is worse than that of testicular primary site, so the survival data of the patients with poor risk in the current study was worse than reported in previously published studies. In contrast, the IGCCCG Update Consortium reported a 5-year OS rate of 96% for good-risk metastatic nonseminomatous GCTs [11] and 95% for seminoma [12]. In our study, the good-risk group demonstrated a similarly high 5-year OS rate of 99.05%. Although this rate is slightly higher than that reported in the IGCCCG update, it is important to note that our sample size is considerably smaller, which may contribute to the observed differences. Nevertheless, our findings are consistent with the expected outcomes for BEP-based regimens, which typically achieve cure rates above 90% in good-risk patients. However, whether the modified BEP regimen is equivalent to the conventional BEP for high-risk patients requires validation through large-scale randomized trials.

Pulmonary toxicity is a well-known effect of bleomycin that presents as pneumonitis, and pulmonary fibrosis may be fatal in 1%–3% of patients administered high doses (> 300 IU) intravenously [13]. Prior poor lung function, a history of smoking, and impaired renal function may predispose patients to developing pulmonary toxicity [14]. A randomized phase III study that randomized patients to receive bleomycin over 72 h demonstrated equivalent outcomes and lung toxicity compared to conventional administration, providing valuable support for the safety of our approach. This study suggests that alternative administration schedules for bleomycin can be as effective and safe as conventional methods. Furthermore, the study highlighted the importance of monitoring for cough and early chest CT scanning to evaluate potential lung toxicity, aligning with our approach to closely monitor clinical symptoms and perform long-term CT follow-up to identify and manage pulmonary toxicity [15]. Our modified BEP regimen also raised concern over the pulmonary toxicity of bleomycin since intensive bleomycin was administered on days 1, 3, and 5. However, strict observation of clinical symptoms and long-term CT follow-up identified only 4 patients (1.46%) with pulmonary toxicity, all of whom recovered fully after treatment.

In addition to pulmonary toxicity, the most common toxicities of the BEP regimen are hematological toxicity and gastrointestinal toxicity, with incidences of febrile neutropenia and vomiting ranging from 7%− 19.4% and 39.3%− 46%, respectively [5, 16, 17]. Moreover, in routine practice, weekly bleomycin administration is prone to poor patient compliance and failure to treat with bleomycin on time, due to toxicity and frequent hospitalization. To reduce the toxicity of the BEP regimen, several studies revised the dose, interval, or duration of bleomycin administration. In a retrospective study of modified-BEP chemotherapy involving a large number of patients with GCT, 15 U bleomycin via IV on day 1 and continuous infusion of 10 U by IV over 12 h on days 1 to 3 found that survival outcome and tumor response were not inferior to those in previously published studies, and no death due to pulmonary toxicity occurred [6]. In a phase II study evaluating paclitaxel, bleomycin, etoposide, and cisplatin (T-BEP) in patients with poor prognosis nonseminomatous germ cell tumor (NSGCT), 30 IU bleomycin was also administered on days 1, 3, and 5. Clinically significant toxicities associated with T-BEP in patients included grade 3–4 febrile neutropenia (33%), and grade 2 pneumonitis (8%); however, all cases were successfully treated with steroids and antibiotics [8]. The administration of modified BEP in the present study with bleomycin given on days 1, 3, and 5 instead of the standard dosing (days 1, 8, and 15) was based on our experience of using BEP-based regimens during the past 2 decades. In our routine clinical practice, weekly administration of bleomycin has been associated with poor compliance and patient inconvenience. However, adjusting the regimen to administer bleomycin on days 1, 3, and 5 of the cycle has enhanced compliance without increasing toxicity.

The primary limitation of our study stems from the inherent challenges associated with retrospective data, including missing clinical and laboratory information, which complicates the acquisition of a reliable toxicity profile. Despite these challenges, we endeavored to systematically review side effect records and follow up with patients whenever possible to compile a convincing toxicity profile. Another limitation is that a small group of patients were treated with bleomycin at standard doses on days 1, 8, and 15 or on days 2, 9, and 16 when receiving the BEP regimen at our center; therefore, it is difficult to compare the modified BEP regimen with the standard BEP approach, which may have compromised the results. However, our high-volume center, treating over 300 patients annually, has approximately 20 years of experience using this modified BEP regimen and has conducted long-term follow-up without significant fatal pulmonary toxicity observed, which adds to the strength of this research. Given these considerations, conducting large-scale clinical trials to validate the efficacy and safety of this modified BEP regimen remains critically important.

In conclusion, with nearly 2 decades of clinical observation and close follow-up, the modified BEP regimen is an effective and safe treatment approach for adult patients with GCTs in the Chinese population, offering greater convenience compared to the standard BEP regimen.

## Data Availability

Data is provided within the manuscript or supplementary information files.
